# High number of chromosomal copy number aberrations inversely relates to t(11;19)(q21;p13) translocation status in mucoepidermoid carcinoma of the salivary glands

**DOI:** 10.18632/oncotarget.17282

**Published:** 2017-04-20

**Authors:** Johannes H. Matse, Enno C.I. Veerman, Jan G.M. Bolscher, C. René Leemans, Bauke Ylstra, Elisabeth Bloemena

**Affiliations:** ^1^ Department of Oral and Maxillofacial Surgery and Oral Pathology VU University Medical Center, Academic Centre for Dentistry Amsterdam (ACTA) Amsterdam, The Netherlands; ^2^ Department of Oral Biochemistry ACTA, University of Amsterdam and VU University, Amsterdam, The Netherlands; ^3^ Department of Pathology, VU University Medical Center, Amsterdam, The Netherlands; ^4^ Department of Otolaryngology, VU University Medical Center, Amsterdam, The Netherlands

**Keywords:** mucoepidermoid carcinoma, t(11;19)(q21;p13) translocation, chromosomal copy number aberrations, MEC

## Abstract

Although rare, mucoepidermoid carcinoma (MEC) is one of the most common malignant salivary gland tumors. The presence of the t(11;19)(q21;p13) translocation in a subset of MECs has raised interest in genomic aberrations in MEC. In the present study we conducted genome-wide copy-number-aberration analysis by micro-array comparative-genomic-hybridization on 27 MEC samples.

Low/intermediate-grade MECs had significantly fewer copy-number-aberrations compared to high-grade MECs (low vs high: 3.48 vs 30; *p* = 0.0025; intermediate vs high: 5.7 vs 34.5; *p* = 0.036). The translocation-negative MECs contained more copy-number-aberrations than translocation-positive MECs (average amount of aberrations 15.9 vs 2.41; *p* =0.04).

Within all 27 MEC samples, 16p11.2 and several regions on 8q were the most frequently gained regions , while 1q23.3 was the most frequently detected loss.

Low/intermediate-grade MEC samples had copy-number-aberrations in chromosomes 1, 12 and 16, while high-grade MECs had a copy-number-aberration in 8p. The most commonly observed copy-number-aberration was the deletion of 3p14.1, which was observed in 4 of the translocation-negative MEC samples. No recurrent copy-number-aberrations were found in translocation-positive MEC samples.

Based on these results, we conclude that MECs may be classified as follows: (i) t(11;19)(q21;p13) translocation-positive tumors with no or few chromosomal aberrations and (ii) translocation-negative tumors with multiple chromosomal aberrations.

## INTRODUCTION

Mucoepidermoid carcinoma (MEC), although rare, is the most common malignant salivary gland neoplasm. According to the WHO, MEC can be classified as low-, intermediate- or high-grade tumors based on the histological parameters necrosis, anaplasia, neural invasion, mitoses and percentage cystic growth [1]. Prognosis of high grade MEC is worse than that of low and intermediate grade tumors [2].

Determining genomic aberrations within MEC has gained interest because aberrations may be used as a classification tool for MEC. Earlier studies found that a subset of MEC carries a t(11;19)(q21;p13) translocation, leading to the *CRTC1-MAML2* fusion gene [3–9]. MECs that harbor the t(11;19)(q21;p13) translocation generally have a more favorable prognosis than translocation negative tumors , irrespective of histological grade [5, 6].

A few studies have investigated the genomic copy number aberrations in MEC using micro-array comparative genomic hybridization (arrayCGH) technique. They have shown that low grade MEC samples, in general had fewer copy number aberrations than high grade MEC samples [7–9]. Furthermore, these studies found that translocation-positive MEC samples had fewer copy number aberrations compared to translocation-negative MEC samples. Both studies reported the loss of 9p21.3 and the gain of 5p15.33 and 8q24.3 regions. Anzick et al [7] found the loss of the 9p21.3, which harbors the CDKN2AB gene, exclusively in translocation-positive MEC samples), whereas Jee et al [8] reported that translocation-negative MEC samples also harbor this genomic aberration. Both studies concluded that the loss of the *CDKN2A/B* genes was associated with an unfavorable prognosis.

Due to the fact that MEC constitutes a group of diverse, non-frequently occurring tumors and considering different copy number aberrations reported in literature, confirmation of these results in another sample set seems to be warranted. Therefore, we have conducted a genomic analysis using arrayCGH to gain insight into chromosomal copy number in MEC. We compared the aberrations with histological grade and translocation status of each sample. Results suggest that two types of MECs can be distinguished: (i) a group of MECs without t(11;19)(q21;p13) translocation with many copy number aberrations (> 6), independent of histological grade, and (ii) a group of MECs with the t(11;19)(q21;p13) translocation with no or a few copy number aberrations (< 6 ) with two exceptions classified as low and intermediate grade.

## RESULTS

### Clinicopathological characteristics

The clinical and histopathological characteristics of patients and tumors in the study are shown in Table 1. The mean age of patients was 48 years (range 9–82). Eighteen tumors originated in the parotid gland and 9 tumors originated in minor salivary gland. Three patients had loco-regional recurrence of the tumor and 3 other patients developed a metastasis. One patient died of the disease. Of the 27 MECs, 17 were classified as low grade, 6 as intermediate grade and 4 as high grade. FISH analysis revealed that 17 of the total 27 MEC samples harbored the t(11;19)(q21;p13) translocation. These comprised 11 of the 17 low grade, 4 of the 6 intermediate grade and 2 of the 4 high grade samples (Table 1).

**Table 1:** Clinicopathological details of the 27 MEC samples

Sample^1^	Sex	Age	Tumor Site^2^	Recurrence/metastasis	t–status^3^
**LG1**	f	9	PG	–/+	–
**LG2**	m	79	MSG	–/–	+
**LG3**	m	45	PG	–/–	–
**LG4**	f	24	PG	–/–	+
**LG5**	f	13	PG	–/–	+
**LG6**	m	34	MSG	–/–	–
**LG7**	m	81	PG	–/–	+
**LG8**	f	42	PG	–/–	+
**LG9**	m	50	PG	–/–	+
**LG10**	f	25	MSG	–/–	+
**LG11**	f	45	PG	–/–	–
**LG12**	f	64	MSG	–/+	+
**LG13**	m	30	MSG	–/–	+
**LG14**	m	57	PG	–/–	+
**LG15**	m	62	PG	–/–	–
**LG16**	f	43	PG	–/–	+
**LG17**	m	51	PG	–/–	–
**IntG1**	m	24	MSG	–/–	+
**IntG2**	f	58	PG	–/–	–
**IntG3**	f	71	MSG	–/+	+
**IntG4**	m	14	PG	–/–	+
**IntG5**	m	58	PG	–/–	–
**IntG6**	f	52	MSG	+/+	+
**HG1**	m	81	PG	–/–	–
**HG2**	m	43	PG	–/–	+
**HG3**	f	82	PG	–/–	+
**HG4**	f	59	MSG	–/+	–

^1^Histological grading: LG, low grade; IntG, intermediate grade; HG, high grade.

^2^Tumor site: PG, parotid gland; MSG, minor salivary gland.

^3^Presence of translocation t(11;19)(q21;p13).

### Genomic profiles in MEC

**Figure 1:**
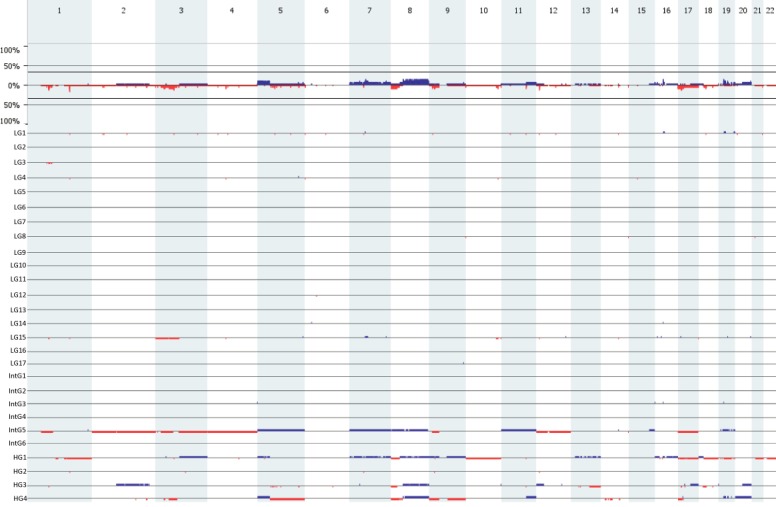
Genome-wide frequency plot (top) The Y-axix represents the percentage of the total group (*n =* 27). Underneath the genome-wide frequency plot, the individual arrayCGH profiles of the of 27 MEC samples (17 low; 6 intermediate; 4 high grade) used in the study. Gains are portrait in blue and losses are protrait in red.

ArrayCGH profiles of 27 MECs are presented in Figure 1. To be sure that we were picking up real copy number aberrations and not static we considered a copy number aberration real when it was being found in at least 3 samples). Using this criteria we found 37 gain and 23 losses (for all copy number alterations see Table 2). The most common copy number aberration was the loss of the 1q23.3 region, which was found in 5 MEC samples.

**Table 2:** Recurring copy number aberrations in 27 MEC samples

Chromosome band	Region coordinates	Region Length (bp)	Event	No. of samples	Candidate genes*
1p31.1	81271921-82317822	1045901	Loss	3	*ADGRL2*
1p31.1	82317822-83535674	1217852	Loss	4	
1p31.1-p22.3	83535674-85092491	1556817	Loss	3	
1q23.3	162789883-163066609	276726	Loss	5	*RGS4*
3p23-p22.2	31951947-38114334	6162387	Loss	3	*MiR-26a-1, CTDSPL*
3p21.1-p14.1	51942767-69002771	17060004	Loss	3	*PBRM1, ADAMTS9*
3p14.1	71138660-71270186	131526	Loss	4	*FOXP1*
5p15.33 - p14.2	0-24691408	24691408	Gain	3	*PDCD6 TRIO*
5p14.1	26738646-26959771	221125	Gain	3	
5p14.1-p13.3	26959771-33389493	6429722	Gain	3	*DROSHA*
5p13.3-p13.2	33389493-36093914	2704421	Gain	3	*ADAMTS12, TARS, RAD1*
5p13.1-q11.1	38884395-47700000	8815605	Gain	3	*DAB2*
5q12.3-q13.1	66116648-67826992	1710344	Loss	3	*PIK3R1*
7p14.1	38969086-40708290	1739204	Gain	3	*POU6F2*
7q11.1-q11.21	59100000-62420609	3320609	Gain	3	
7q11.21-q11.23	62420609-71886202	9465593	Gain	3	*AUTS2*
7q34	140141708-140795070	653362	Gain	3	*BRAF*
8p23.3-p21.2	0-24154814	24154814	Loss	3	*DEFA1, DEFB1, DLC1, MTUS1*
8q11.1-q11.21	45200000-48882980	3682980	Gain	4	
8q11.21-q11.22	48882980-51942456	3059476	Gain	3	*SNAI2*
8q12.1	52901077-56253836	3352759	Gain	3	
8q12.1	56253836-57369226	1115390	Gain	4	*PLAG1*
8q12.1	57369226-59262605	-1893379	Gain	3	
8q12.1-q12.2	59262605-61867310	2604705	Gain	4	*CYP7A1, SDCBP*
8q12.2-q12.3	61867310-63949108	2081798	Gain	3	
8q12.3-q13.1	63949108-66631815	2682707	Gain	4	*CYP7B1*
8q13.1-q13.2	66631815-69704074	3072259	Gain	3	
8q13.2-q13.3	69704074-71870918	2166844	Gain	4	*SULF1*
8q13.3-q21.12	71870918-79997004	8126086	Gain	4	
8q21.12-q21.13	79997004-82856098	2859094	Gain	3	*FABP5*
8q21.13-q21.2	82856098-85047238	2191140	Gain	4	
8q21.2-q21.3	85047238-87786949	2739711	Gain	3	*WWP1*
8q21.3	87786949-90946365	3159416	Gain	4	
8q21.3	90946365-92054675	1108310	Gain	3	
8q21.3-q22.3	92054675-102317166	10262491	Gain	4	*RUNX1T1, CDH17, TP53INP1*
8q22.3	102317166-104094744	1777578	Gain	3	
8q22.3-q23.1	104094744-107148474	3053730	Gain	4	*BAALC, CTHRC1*
8q23.1	107939667-109558687	1619020	Gain	3	*ANGPT1*
8q23.1-q24.23	109558687-139487231	29928544	Gain	4	*HAS2, TNFRSF11B, MYC*
8q24.23-q24.3	139487231-140865305	1378074	Gain	3	*TRAPPC9*
8q24.3	140865305-146274826	5409521	Gain	4	*PTK2, MAFA, MAPK15*
9p23-p22.3	13398392-14364288	965896	Loss	3	
9p21.3	20451378-20600059	148681	Loss	3	
9p21.3	21851680-23259683	1408003	Loss	3	*CDKN2A, CDKN2B, MTAP*
11q21	95420121-95735922	315801	Loss	3	*MAML2*
12p13.2	11187958-11860096	672138	Loss	4	*ETV6, PRB1*
16p11.2	31753818-33345523	1591705	Gain	4	
17p13.3	0-197784	197784	Loss	3	
17p13.3-p13.1	927102-7097922	6170820	Loss	3	*PLD2, MiR134*
17p13.1-p12	7771326-11457982	3686656	Loss	3	*ALOX15B*
17p12	11457982-12504361	1046379	Loss	4	*MAP2K4*
17p12	12504361-13787450	1283089	Loss	3	*ELAC2*
17p12	13787450-14819919	1032469	Loss	4	
17p12-p11.2	14819919-17694955	2875036	Loss	3	
17p11.2-q11.1	19523822-22200000	2676178	Loss	3	*MAP2K3*
18q12.1	26913332-28779980	1866648	Loss	3	*DSC3*
19p12	19860022-20054862	194840	Gain	3	
19p12-q11	20054862-28500000	8445138	Gain	3	
19q12	34912078-36065910	1153832	Gain	3	
20q11.1-q13.33	27100000-59628718	32528718	Gain	3	*WISP2, MAFB, MIR296*

*gene location according to UCSC Genome Browser on Human Feb. 2009 (GRCh37/hg19) Assembly.

**Figure 2:**
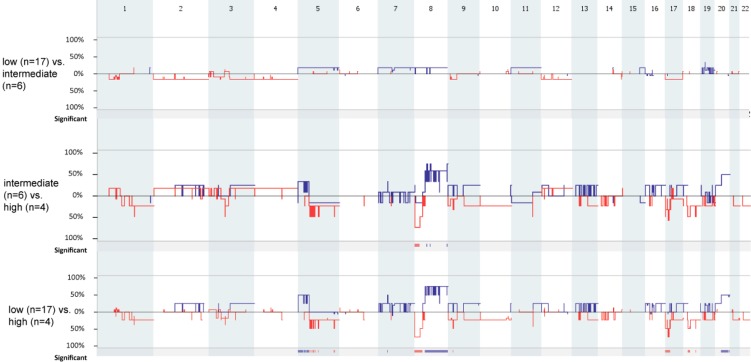
Genome-wide comparison between low (*n* = 17) vs. intermediate (*n* = 6), intermediate (*n* = 6) vs. high (*n* = 4), and low (*n* = 17) vs. high (*n* = 17) grade MEC samples. Gains are portrait in blue and losses are portrait in red Regions of *p <* 0.05 are marked by horizontal bars of gains (blue) and losses (red) on the significance track.

Analysis based on histological grading (Figure 2) revealed that there was no differences between the number of copy number alterations between low and intermediate grade MEC samples (*p =* 0.763) and between intermediate and high grade MEC samples (*p =* 0.099). Therefore, we combined the low and intermediate grade MEC samples to form a low/intermediate grade MEC group.

Low/intermediate grade MEC samples had statistically significantly fewer copy number aberrations compared to high grade MEC samples (mean values: low/intermediate grade vs high grade: 3.48 vs 30, *p =* 0.0025). The deletion of 1p31.1 (containing *ADGRL2*), 1p31.1-p22.3 and 12p13.2 (containing *ETV6*), and the gain of 16p11.2 were exclusively found in the low/intermediate grade MEC samples, while the loss of 8p23.3-p12 (containing *DEFB1*, *DLC1*, *MTUS1*) was exclusively found in high grade MEC samples (Table 3).

**Table 3:** Recurrent copy number aberrations exclusively found in low/intermediate grade (*n* = 23) or in high grade MEC samples (*n* = 4)

Chromosome band	Region coordinates	Region Length (bp)	Event	No. of samples	Present in LG/IntG or HG1	Candidate genes*
1p31.1	81271921-82317822	1045901	Loss	3	*LG/IntG*	*ADGRL2*
1p31.1-p22.3	83535674-85092491	1556817	Loss	3	*LG/IntG*	
8p23.3-p21.2	0-24154814	24154814	Loss	3	*HG*	*DEFB1, DLC1, MTUS1*
12p13.2	11187958-11860096	672138	Loss	3	*LG/IntG*	*ETV6*
16p11.2	31753818-33345523	1591705	Gain	3	*LG/IntG*	

^1^LG/IntG, low/intermediate grade MEC; HG, high grade MEC.

*gene location according to UCSC Genome Browser on Human Feb. 2009 (GRCh37/hg19) Assembly.

**Figure 3:**
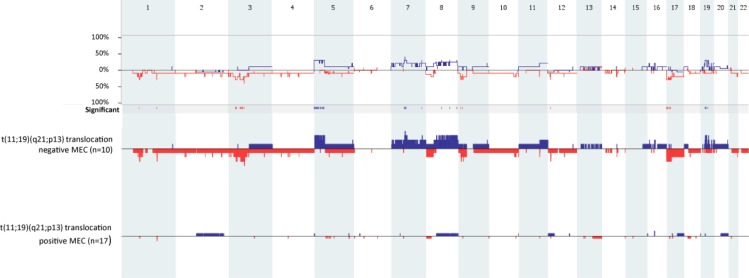
Genome-wide comparison between t(11;19)(q21;p13) translocation-positive (*n* = 17) and t(11;19)(q21;p13) translocation-negative MEC samples (*n* = 10) Gains are portrait in blue and losses are portrait in red. Regions of *p <* 0.05 are marked by horizontal bars of gains (blue) and losses (red) on the significance track.

Translocation-positive MEC contained fewer copy number aberrations than translocation-negative tumors (Figure 3) (mean values translocation-positive vs translocation-negative: 2.41 vs 15.9, *p =* 0.04). In total 22 copy number aberrations were found (11 loss and 11 gains), which were all found to be exclusive for translocation-negative MEC samples (see Table 4 for all copy number aberrations that were present in 3 or more translocation-negative MEC samples). The most common copy number aberration detected was the loss of 3p14.1 (containing *FOXP1*), which was found in 4 translocation-negative MEC samples (LG1, LG15, HG1 and HG4).

**Table 4:** Recurring copy number aberrations exclusively found in MEC samples without the t(11;19)(q21;p13) translocation (*n* = 10)

Chromosome band	Region coordinates	Region length (bp)	Event	No. of samples	Candidate gene(s)*
1p31.1	81271921–82317822	1045901	Loss	3	*ADGRL2*
3p23-p22.2	31951947–38114334	6162387	Loss	3	*MiR-26a-1, CTDSPL*
3p14.1	71138660–71270186	131526	Loss	4	*FOXP1*
5p15.33-p14.2	0–24691408	24691408	Gain	3	*PDCD6, TRIO*
5p14.1	26738646–26959771	221125	Gain	3	
5p14.1-p13.3	26959771-33389493	6429722	Gain	3	*DROSHA*
5p13.3-p13.2	33389493–36093914	2704421	Gain	3	*ADAMTS12, TARS, RAD1*
5p13.1-q11.1	38884395–47700000	8815605	Gain	3	*DAB2*
7q11.1-q11.21	59,100,000–62,420,609	3320609	Gain	3	
7q11.21-q11.23	62420609–71886202	9465593	Gain	3	*AUTS2*
7q34	140141708–140795070	653362	Gain	3	*BRAF*
8q24.23-q24.3	139487231–140865305	1378074	Gain	3	*TRAPPC9*
9p23-p22.3	13398392–14364288	965896	Loss	3	
9p21.3	21851680–23259683	1408003	Loss	3	*CDKN2A, CDKN2B, MTAP*
17p13.3-p13.1	927102–7097922	6170820	Loss	3	*PLD2, MiR134*
17p13.1-p12	7771326–11457982	3686656	Loss	3	*ALOX15B,*
17p12	11457982–12504361	1046379	Loss	3	*MAP2K4*
17p12	12504361–13787450	1283089	Loss	3	*ELAC2,*
17p12-p11.2	14819919–17694955	2875036	Loss	3	
17p11.2-q11.1	19523822–22200000	2676178	Loss	3	*MAP2K3*
19p12-q11	20054862–28500000	8445138	Gain	3	
19q12	34912078–36065910	1153832	Gain	3	

*gene location according to UCSC Genome Browser on Human Feb. 2009 (GRCh37/hg19) Assembly.

To demonstrate that the translocation-negative tumors are bona fide MEC, especially those with *EVT6* loss, *PLAG1* gain, and those derived from minor salivary glands (Supplementary Table 1), additional histologic pictures including immunohistochemical profiles using p63, S100 and AR (Supplementary Table 2) are shown in Supplementary Figures 1 and 2.

## DISCUSSION

Mucoepidermoid carcinomas are salivary gland tumors with a variable histopathological differentiation. They have an unpredictable clinical outcome, which poses significant diagnostic and therapeutic challenges. Analysis of genomic aberrations may help in the classification of these tumors, but large scale analysis of the genomic imbalance in MEC is hampered because of its rather low frequency of occurrence. There are several arrayCGH based studies described as yet [7–9], using different spatial resolution, with different cut-offs for specific genetic aberration for all or for a subset of MEC. To us, this warranted further analysis of the genomic imbalance in an additional set of 27 well documented MECs.

In the present study, copy number aberrations were found in 14 out of 27 MEC samples (Table1). Based on the amount of copy number aberrations, two groups of MEC can be distinguished. One group with no or few copy number aberrations (6 or less), and another with multiple copy number aberrations (19 or more); the latter being about 22% of the total number of MEC samples. MEC samples harboring multiple copy number aberrations were found amongst all three histological grades.

With the exception of one sample (HG3), most of the MEC with multiple copy number aberrations were translocation-negative, while translocation-positive MEC samples had mostly 6 or less copy number aberrations. These findings confirmed the consensus in literature and may explain why t(11;19)(q21;p13) translocation-positive MEC have a more favorable survival outcome compared to transformation negative MECs, which are characterized by chromosomal instability [7–9].

Comparison with previous studies [7–9] underscores that MEC is extremely diverse with respect to copy number aberrations. For instance, the most frequently detected loss in the present set of MECs, 1q23.3 (containing *RGS4*) in 5 of the 27 MECs (Table 2), was not reported in the other studies, whereas the most frequently detected loss reported by Jee et al [8], 18q12.2-qter, was found only once in our sample set. Similarly, the majority of most frequently detected gains found in this study (Table 2) did not correspond with earlier research [8]. Only a set of small regions that were gained in chromosome 8 corresponded with a larger gain 8q11.1-q12.2 described by Jee et al [8].

Only one region found in our study, the lost region 9p21.3, was recorded to be by earlier studies [7, 8, 9]. The loss of 9p21.3, containing *CDNK2A/B,* initially suggested this occurrence to be specific to translocation-positive MEC [7], but later was also found in translocation-negative MEC [8]. In both studies, the loss of *CDKN2A/B* was associated with an unfavorable prognosis. Zhang et al [12] found that the region 9p21.3 was also deleted in adenoid cystic carcinoma and salivary duct carcinoma. Furthermore, the deletion of 9p21.3 is a frequent oncogenic event observed in head and neck squamous cell carcinomas, and in lung cancer [10–12].

The regions 8q23.1-q24.23 (myelocytomatosis oncogene (*MYC*)) and 8q24.3 (protein tyrosine kinase 2 (*PTK2*)) were gained in 4 samples, which all had multiple copy number aberrations. *PTK2* has been shown to be gained in prostate, gastric, colorectal cancers and in salivary adenoid cystic carcinoma [13–16]. *MYC* is overexpressed in nearly 50% of all human tumors [17, 18], resulting in the aberrant expression of *MYC* target genes. The t(11;19)(q21;p13) fusion protein can bind and activate *MYC,* leading to cellular transformation via functional complementation of CREB and *MYC* transcription networks [19, 20]. In our study however, only one of the 4 samples with a gain of 8q23.1-q24.23 harbored the translocation.

Although some chromosomal aberrations may suggest the involvement of certain genes, the general instability of these malignancies may also be of importance for MEC development. Therefore, clinically, the instability itself should be taken as a marker rather than specific (onco)genes that are gained or lost in specific samples.

In conclusion, in this study we showed that salivary gland MEC may be classified as follows: (i) MEC with no or few chromosomal aberrations, which are in general positive for the t(11;19)(q21;p13) translocation, and (ii) MEC with multiple genomic imbalances, which are in general t(11;190(q21;p13) translocation negative. This implies that there are different oncogenic pathways within MEC, in which either the fusion-gene or the loss of genetic instability plays a role in the underlining pathologic process.

## MATERIALS AND METHODS

### Samples

Formalin-fixed paraffin-embedded MEC samples and matched healthy salivary gland samples were retrieved from the archives of the Department of Pathology, VU University medical center, Amsterdam, The Netherlands. All tumors were surgically removed between 1984 and 2012. Hematoxylin and eosin stained sections (4 µm) were reviewed by an experienced pathologist (EB) who confirmed the original diagnosis and graded the tumors. Twenty-seven cases of which there was no doubt about the diagnosis were used for this study. All parotid tumors in patients with a previous history of cutaneous squamous cell carcinoma in which there was the slightest doubt about the classification of the parotid tumor were excluded from the study. Clinicopathological details are described in Table 1. The design of the study adhered to the code for proper secondary use of human tissue established by the Dutch Federation of Biomedical Scientific Societies (http://www.federa.org) [21].

### DNA isolation

DNA was isolated as previously described [22]. Briefly, 6 sections of 10 μm were deparaffinized, macro-dissected and incubated with 1M sodium thiocyanate at 38°C, for 16 h, followed by a proteinase K treatment at 55°C for another 16 h. DNA was isolated using the QIAmp DNA micro-kit (Qiagen, Hilden, Germany). Purity and quantity of the DNA samples was measured using a Nanodrop 2000 spectrophotometer (Thermo Scientific, Waltham, USA).

### ArrayCGH

ArrayCGH was performed as described previously [23]. Although FFPE is not the most ideal material for aCGH analyses, we have over the years built a large amount of experience herewith generating good quality data [23]. Equal amounts (500 ng) of DNA from MEC samples and from matching normal salivary gland tissue of each patient individually were labeled with cyanine 3′-dUTP (Cy3) and cyanine 5′-dUTP (Cy5) nucleotides (Enzo Life Sciences, Farmingdale, NY, USA). Free nucleotides were removed using the MinElute PCR Purification Kit (Qiagen). Oligonucleotide arrayCGH was performed using the SurePrint G3 Human CGH Microarray Kit, containing 180880 *in situ* synthesized 60-mer oligonucleotides representing 169793 unique chromosomal locations evenly distributed over the genome (space ∼17kb) and 4548 additional unique oligonucleotides, located at 238 of the Cancer Census genes (4x180K array, Agilent Technologies, Palo Alto, CA, USA). The exact array design can be found online in the Gene Expression Omnibus (GEO) GPL8687 (http://www.ncbi.nlm.nih.gov/geo). The data are accessible through GEO number GSE87353. Segment values were converted to calls by setting thresholds corresponding to 20% of the tumor cells with that copy number aberration: this percentage converts to a log2 ratio of > 0.1375 for gains and < –0.1520 for losses and all values in between are called normal copy number. Values above the 0.1375 threshold were called gains, values below the –0.1520 threshold were called losses. Although the threshold for detection of aberration calls is low for FFPE material, this was nevertheless chosen upon visual inspection of all profiles as the optimal balance between background and detection of real copy number aberrations. Nevertheless, for analyses, we have focused on recurrent copy number aberrations that occurred in at least three tumors. The log2 ratio threshold for high copy number amplification and homozygous deletion were 1.0 and -1.0, respectively. The data were analyzed using Nexus, in which the significance threshold was set at *P* < 0.05.

To make sure that the detected copy number alterations were real and not background static, we considered a copy number real when it was present in 3 or more MEC samples.

### FISH analysis

For detection of the translocation in MEC samples, fluorescence *in situ* hybridization (FISH) analysis was carried out on 4 μm tissue sections according to the manufacturer’s protocol, using ZytoLight ^®^ SPEC MAML2 Dual Color Break Apart Probe (ZytoVision Ltd, Bremerhaven, Germany). The MAML2 Dual Color Break Apart Probe can detect rearrangements involving the MAML2 gene irrespective of the fusion partner (including the CRTC3-MAML2 fusion). The nuclei were counterstained with 4′,6-diamidino-2-phenylindole (DAPI), diluted in Vectashield, and samples were evaluated by fluorescence microscopy (ZyGreen: excitation 503 nm, emission 528 nm; ZyOrange: excitation 547 nm, emission 572 nm). Cells without the t(11;19)(q21;p13) translocation show fused green and red signals, typically resulting in a yellow signal. Translocation-positive cells exhibit fused green and red, as well as separated red and green signals, or split signal (Figure 1). A MEC sample was considered positive for the t(11;19)(q21;p13) translocation when the split signal was identified in at least 10 out of 100 cells.

### Statistical analysis

Differences between the presence of copy number aberrations in low grade, intermediate grade and high grade MEC, translocation-positive and translocation-negative MEC samples were determined using the Mann-Whitney test. A two-sided *P*-value of ≤ 0.05 was considered to be statistically significant. Statistical analyses were performed with the use of the Statistical Package for the Social Sciences (SPSS version 20.0).

## SUPPLEMENTARY MATERIALS FIGURES AND TABLES


