# Supplementary material for: High number of chromosomal copy number aberrations inversely relates to t(11;19)(q21;p13) translocation status in mucoepidermoid carcinoma of the salivary glands
Source: Oncotarget. 2017 Apr 20;8(41):69456–64. doi: 10.18632/oncotarget.17282 (PMC5642491; doi:10.18632/oncotarget.17282)
Supplement: Supplementary file 1 [file oncotarget-08-69456-s001.pdf]

High number of chromosomal copy number aberrations inversely relates to t(11;19)(q21;p13) translocation status in mucoepidermoid carcinoma of the salivary glands

Supplementary Materials

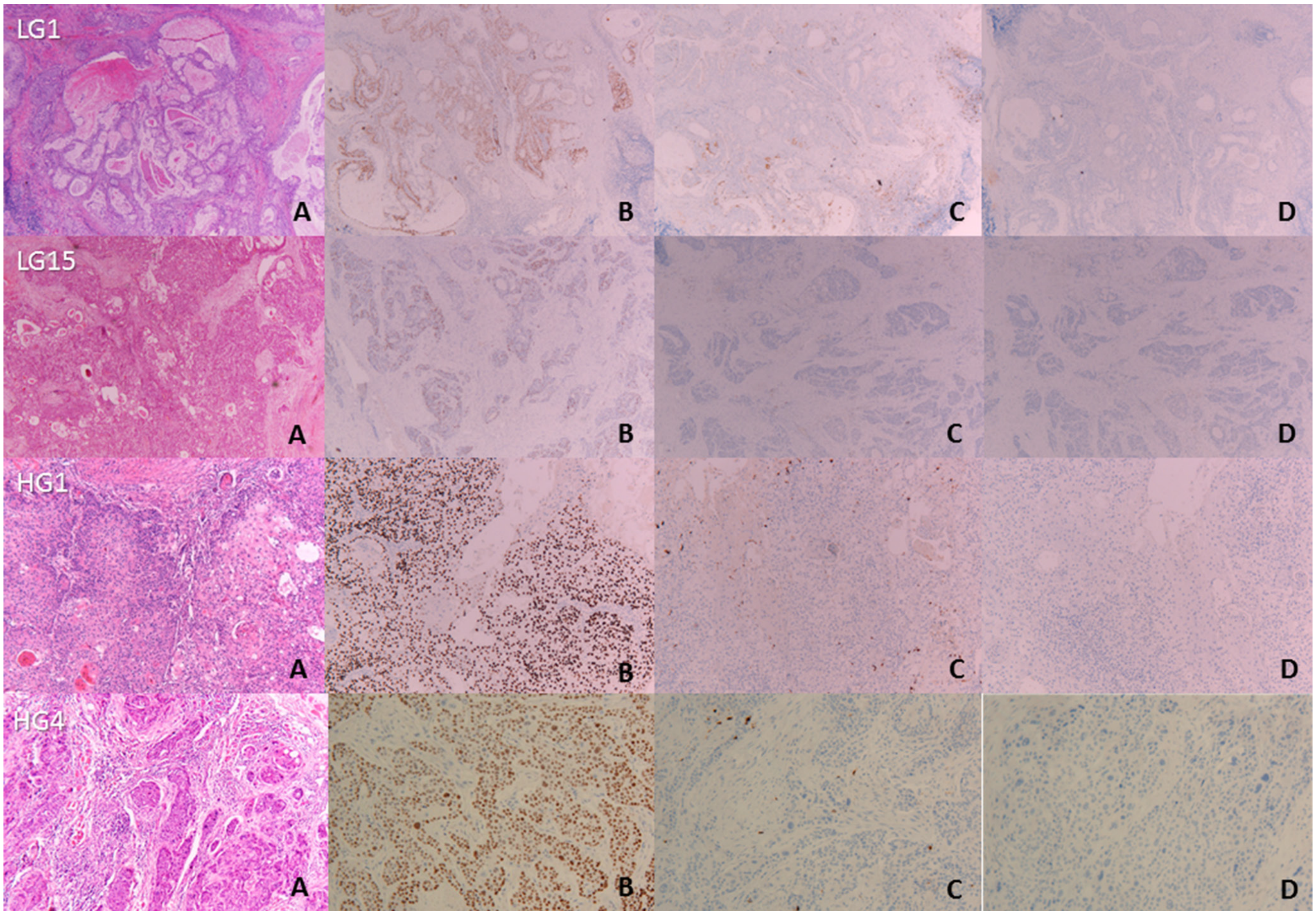

Figure 1: Histology of translocation-negative mucoepidermoid carcinomas. Row 1: tumor LG1. Row 2: tumor LG15. Row 3: tumor HG1. Row 4: tumor HG4. (**A**) Staining H&E; (**B**) Immunohistochemical staining for p63; (**C**) Immunohistochemical staining for S100; (**D**) Immunohistochemical staining for AR. (Specifications of immunohistochemistry in Supplementary Table 2.)

Supplementary Table 1: Translocation negative MEC, with *EVT6* loss, *PLAG1* gain or from minor salivary glands with reference to the Supplementary Figures depicting these tumors

Tumor	translocation	*EVT* loss	*PLAG1* gain	Minor salivary gland	Supplementary Figure
LG1	neg	yes	no	no	1
LG6	neg	no	no	yes	2
LG15	neg	yes	no	no	1
IntG5	neg	yes	yes	no	2
HG1	neg	no	yes	no	1
HG4	neg	no	yes	yes	1

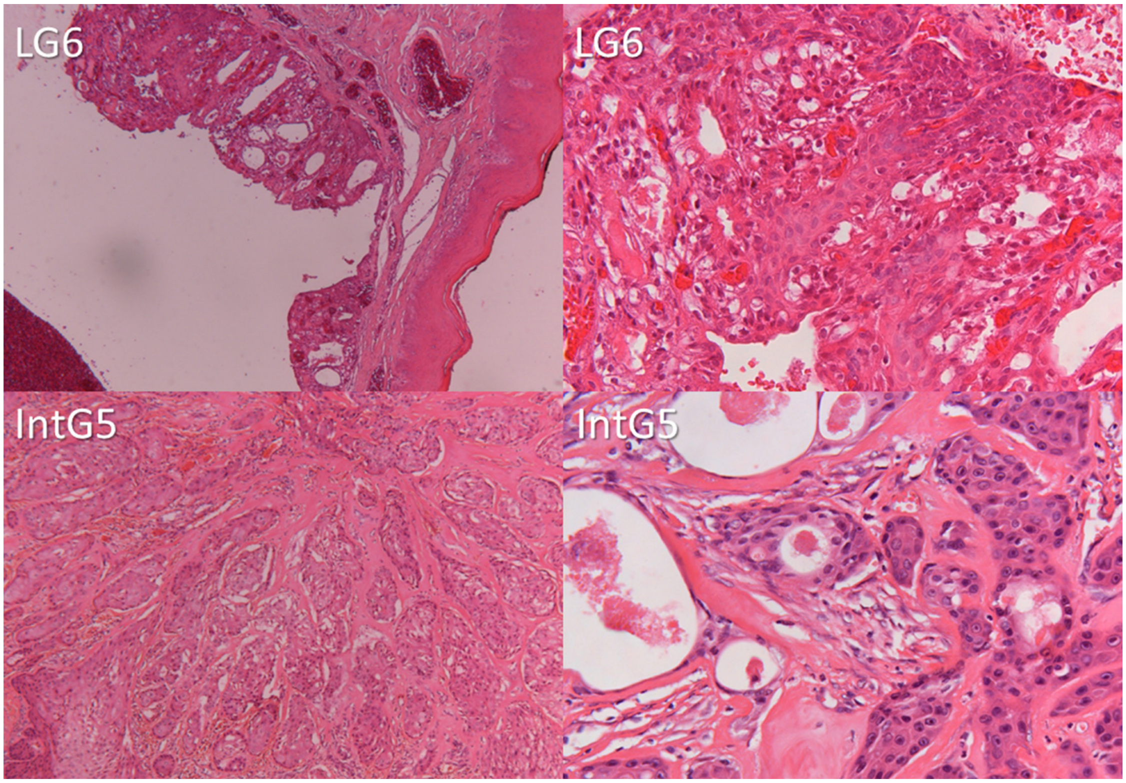

Figure 2: Histology of translocation-negative mucoepidermoid carcinomas. Row 1: tumor LG6. Row 2: tumor IntG5.

Supplementary Table 2: Specifications of antibodies used for immunohistochemistry

Antibody	Firm	Clone	Species	Dilution	Detection	Retrieval
P63	Immunologics (Klinipath)	4A4	rabbit	1:50	Optiview	CC1
S100	DAKO	poly	rabbit	1:5000	Optiview	CC1
AR	Spring Bioscience	SP107	mouse	1:600	Optiview	CC1
